# Use of Endo GIA™ With Tri-staple™ and Local Muscle Flaps in Neopharyngeal Reconstruction Following Salvage Total Laryngectomy

**DOI:** 10.7759/cureus.94292

**Published:** 2025-10-10

**Authors:** Ömer Bayır, Musa Alper Batı, Serhat Tokgöz, Güleser Saylam, Mehmet H Korkmaz

**Affiliations:** 1 Otolaryngology - Head and Neck Surgery, Ankara Etlik City Hospital, Ankara, TUR; 2 General Surgery, Ankara Etlik City Hospital, Ankara, TUR; 3 Otolaryngology - Head and Neck Surgery, Faculty of Medicine, Lokman Hekim University, Ankara, TUR; 4 Otolaryngology - Head and Neck Surgery, Private Clinic, Ankara, TUR

**Keywords:** laryngeal cancer, laryngectomy, salvage therapy, surgical flap, surgical stapler

## Abstract

Salvage total laryngectomy (STLx) is associated with higher complication rates than primary procedures, particularly in patients with a history of radiation therapy, where delayed wound healing and pharyngocutaneous fistula remain major challenges. Commonly recommended approaches, such as pedicled or free tissue flaps, may reduce complications but often prolong operative time, enlarge the surgical field, and add morbidity. In this technical report, we describe a neopharyngeal reconstruction technique using Endo GIA™ with Tri-Staple™ Technology (Covidien, North Haven, Connecticut), reinforced with local muscle flaps, as an alternative in STLx. After laryngeal mobilization, the neopharynx was closed with a 60-mm stapler, with an additional cartridge when necessary, and reinforced with submucosal and cricopharyngeal sutures. Bilateral sternocleidomastoid sternal heads and strap muscles were rotated over the staple line to provide vascularized support. This approach offered secure closure, shortened operative time, minimized reconstructive complexity, and avoided donor site morbidity, while the local muscle reinforcement enhanced vascularity and reduced the risk of fistula formation in high-risk irradiated patients. We believe that stapler-assisted neopharyngeal closure combined with local muscle flaps represents a practical and effective strategy for STLx, with the potential to lower complication rates, facilitate recovery, and serve as a valuable alternative to more extensive reconstructive procedures.

## Introduction

Salvage total laryngectomy (STLx) is often required for recurrent or persistent disease after chemoradiotherapy. Compared with primary procedures, salvage cases are associated with significantly higher complication rates, largely due to the hypovascular, hypocellular, and hypoxic nature of irradiated tissues [1]. The most common and clinically relevant complication is pharyngocutaneous fistula (PCF), with reported rates ranging from 11% to 58% in salvage settings [2].

Several risk factors have been identified for PCF, including chronic obstructive pulmonary disease, low preoperative hemoglobin, blood transfusion, prior radiotherapy or chemoradiotherapy, advanced tumor stage, hypopharyngeal involvement, positive surgical margins, and neck dissection [3]. These findings underscore the importance of reliable closure and reinforcement strategies.

Vascularized tissue reinforcement is a well-established approach. Pectoralis major myofascial onlay flaps have been shown to decrease PCF rates in salvage patients compared with primary closure [4]. However, such flaps can prolong surgery and increase donor site morbidity. Local flaps, such as the sternocleidomastoid and strap muscles, provide vascularized coverage with fewer disadvantages [5].

Stapler-assisted closure of the neopharynx has emerged as an alternative to hand suturing. Evidence from meta-analyses and randomized trials indicates that stapler closure significantly reduces PCF rates, shortens operative time and hospital stay, and facilitates earlier oral feeding [6-8]. Importantly, stapler closure has demonstrated comparable oncologic safety to conventional suturing.

In this report, we describe a hybrid technique of Endo GIA™ with Tri-Staple™ (Covidien, North Haven, Connecticut) stapler closure reinforced with local muscle flaps, aiming to combine the advantages of mechanical stapling with the vascular support of locoregional tissue.

## Technical report

In the operating room, standard monitoring, including electrocardiography, noninvasive blood pressure measurement, and pulse oximetry, was initiated. After preoxygenation with 100% oxygen, general anesthesia was induced using intravenous propofol (2 mg/kg) and fentanyl (1 mcg/kg). Once effective mask ventilation was confirmed, rocuronium (0.6 mg/kg) was administered to facilitate neuromuscular blockade. The patient was ventilated with 100% oxygen for three minutes before endotracheal intubation. Patients with hypopharyngeal extension and base of tongue involvement are not suitable for this technique, as they may require free flaps or pedicled musculocutaneous flaps.

Following antisepsis with povidone-iodine, the patient was positioned supine with a shoulder roll to optimize neck extension. The head and neck were aligned in a neutral midline position, and nonsurgical areas were carefully draped under sterile conditions.

**Figure 1:**
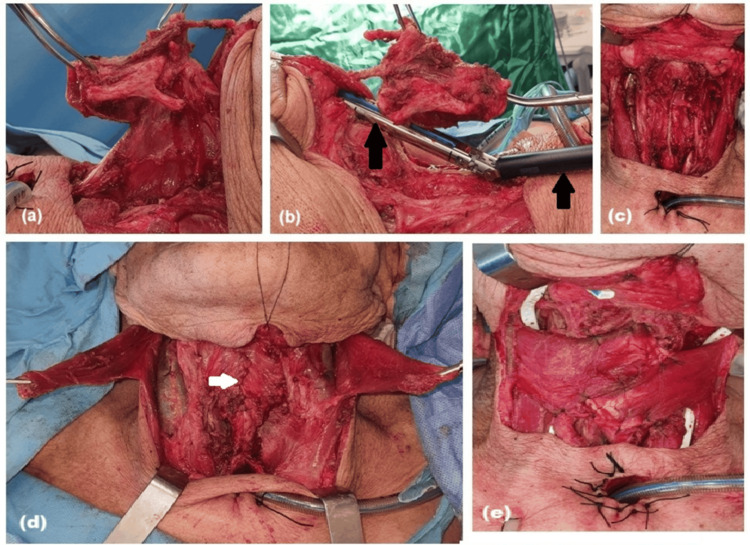
Stepwise procedure for salvage total laryngectomy with neopharyngeal reconstruction (a) Mobilization of the strap muscles, division of the thyroid isthmus, and dissection of the hypopharyngeal mucosa while preserving the lingual surface of the epiglottis. (b,c) Placement and firing of a 60-mm Endo GIA™ with Tri-Staple™ stapler parallel to the esophagus and pharynx, simultaneously closing and separating the pharynx and larynx. The black arrows indicate the Endo GIA™ with Tri-Staple™ stapler. (d) Closure of a second submucosal layer and reinforcement with cricopharyngeus muscle suturing. The white arrow indicates the closure line manually performed with 3/0 Vicryl. (e) Rotation of the sternal heads of both sternocleidomastoid muscles over the reconstruction line to provide additional coverage

The strap muscles were separated from the hyoid bone and linea alba while preserving their integrity, and opened laterally. The thyroid isthmus was divided. The trachea was mobilized and transected full-thickness, and a permanent stoma was created. The larynx was then mobilized from the posterior aspect of the cricoid cartilage to the arytenoids. The hyoid bone was skeletonized, and dissection continued inferiorly to the epiglottis. The epiglottis was dissected while preserving the lingual mucosal surface. The thyroid cartilage was freed, and the hypopharyngeal mucosa was dissected (Figure 1a).

The epiglottic cartilage was grasped with a clamp and pulled outward to ensure correct stapler positioning and to prevent inadvertent inclusion of the epiglottis within the stapling line. The larynx was elevated, and a 60-mm Endo GIA™ with Tri-Staple™ stapler (with an additional 45 mm if needed) was placed longitudinally, parallel to the esophagus and pharynx, and close to the posterior surface of the larynx. The stapler was fired, simultaneously closing the pharynx and larynx with three rows of staples and separating them (Figures 1b, 1c).

A feeding tube was inserted, and oral saline was used to check for mucosal leaks. A second submucosal layer was closed continuously with 3/0 Vicryl (Ethicon, Bridgewater, New Jersey), and the cricopharyngeus muscle was sutured as a third reinforcing layer with 3/0 Vicryl (Figure 1d). The sternal heads of both sternocleidomastoid muscles were transected, rotated over the reconstruction line, and sutured in place (Figure 1e). If the internal jugular vein and carotid artery remained exposed, the strap muscles were rotated laterally to cover them. If already covered, the strap muscles were sutured directly over the reconstruction line. The tracheal stoma was created in primary surgery. Drains were placed, and the operation was completed.

In the postoperative period, the patient was monitored in the intensive care unit for 24 hours with hourly evaluation of fluid balance, vital signs, and drain output. After 24 hours, the patient was transferred to the Head and Neck Surgery Ward. Monitoring of vital signs, wound status, and stoma condition continued.

Tracheostoma sutures were removed gradually from postoperative day 10, with two to three sutures taken out daily to minimize tissue trauma. Enteral feeding via nasogastric tube was maintained for 14 days to ensure adequate nutrition while protecting the surgical site. On postoperative day 14, oral feeding was initiated with a soft diet. Once daily oral intake exceeded 1,500 cc, the nasogastric tube was removed. This technique reduces complication rates without requiring tissue transfer, while shortening operative time and avoiding additional morbidity.

## Discussion

STLx presents considerable challenges due to the compromised vascularity of irradiated tissues and the high incidence of complications. PCF remains the most significant problem, as it prolongs hospitalization, delays adjuvant therapy, and increases morbidity and mortality. Comparative studies confirm that perioperative complications are more frequent in salvage cases than in primary procedures, even though long-term survival outcomes may be similar [1].

Stapler-assisted closure has consistently demonstrated advantages over hand suturing. A systematic review and meta-analysis showed lower PCF rates with stapler closure compared with conventional techniques, along with reduced operative time and hospital stay [6]. Randomized clinical evidence further confirmed these benefits, demonstrating fewer fistulas, shorter surgeries, improved swallowing outcomes, and no adverse impact on oncologic safety [7]. A recent cohort study also reported substantially lower fistula rates in stapler groups compared with hand-sutured closure [8].

Despite these advantages, stapling alone may not fully offset the detrimental effects of prior radiotherapy. Therefore, reinforcement with vascularized tissue remains an important adjunct. Pectoralis major myofascial flaps have been shown to significantly reduce fistula formation in salvage cases [4]. However, their use can increase morbidity, which has led to growing interest in local muscle flaps. Sternocleidomastoid and strap muscles provide well-vascularized coverage directly at the staple line without the need for distant tissue harvest [5].

The hybrid approach described here integrates stapler-assisted closure with local muscle reinforcement. The stapler provides a uniform, rapid, and reliable mechanical seal, while the muscle flaps enhance vascularity and protect the staple line. This combination directly addresses the two major determinants of fistula formation: closure integrity and tissue vascularization. By reducing fistula risk and operative time while avoiding donor site morbidity, this method represents a practical and effective alternative in STLx.

## Conclusions

STLx in previously irradiated patients remains a challenging procedure due to impaired wound healing and the high incidence of PCF. Our technical approach, combining stapler-assisted neopharyngeal closure with local muscle flap reinforcement, addresses two critical factors: reliable mechanical closure and improved vascularity at the reconstructed site. This hybrid technique not only reduced reconstructive complexity and operative time but also avoided the additional morbidity of distant tissue transfer. By reinforcing the staple line with well-vascularized local muscles, the risk of fistula formation may be further mitigated in high-risk salvage cases. Based on our experience, we believe that this method represents a practical, effective, and safe alternative to more extensive reconstructive procedures, with the potential to improve postoperative outcomes and recovery. Further prospective and comparative studies are warranted to validate these findings and establish this approach as a standard adjunct in STLx.

## References

[1] Sullivan CB, Ostedgaard KL, Al-Qurayshi Z, Pagedar NA, Sperry SM (2020). Primary laryngectomy versus salvage laryngectomy: a comparison of outcomes in the chemoradiation era. Laryngoscope.

[2] Putten L, Bree R, Doornaert PA (2015). Salvage surgery in post-chemoradiation laryngeal and hypopharyngeal carcinoma: outcome and review. Acta Otorhinolaryngol Ital.

[3] Dedivitis RA, Aires FT, Cernea CR, Brandão LG (2015). Pharyngocutaneous fistula after total laryngectomy: systematic review of risk factors. Head Neck.

[4] Gilbert MR, Sturm JJ, Gooding WE, Johnson JT, Kim S (2014). Pectoralis major myofascial onlay and myocutaneous flaps and pharyngocutaneous fistula in salvage laryngectomy. Laryngoscope.

[5] Ibrahim SG, Wahba BM, Elbatawi AM, Eltelety AM (2017). Sternocleidomastoid flap augmentation of the pharyngeal closure after total laryngectomy. Eur Arch Otorhinolaryngol.

[6] Chiesa-Estomba CM, Mayo-Yanez M, Palacios-García JM (2022). Stapler-assisted pharyngeal closure after total laryngectomy: a systematic review and meta-analysis. Oncol Ther.

[7] Mandor EA, Ebada HA, El-Fattah AM, Kamal E, Baz H, Tawfik A (2024). Stapler versus conventional pharyngeal repair after total laryngectomy: a randomized clinical trial. Eur Arch Otorhinolaryngol.

[8] Galazka A, Stawarz K, Bienkowska-Pluta K, Paszkowska M, Misiak-Galazka M (2025). Closure techniques for esophageal reconstruction after total laryngectomy and their impact on fistula formation. World J Clin Oncol.

